# Skin Temperature in Parkinson's Disease Measured by Infrared Thermography

**DOI:** 10.1155/2020/2349469

**Published:** 2020-07-25

**Authors:** Mathias Møller Purup, Karoline Knudsen, Pall Karlsson, Astrid Juhl Terkelsen, Per Borghammer

**Affiliations:** ^1^Department of Nuclear Medicine and PET, Aarhus University Hospital, Aarhus, Denmark; ^2^Danish Pain Research Center, Aarhus University Hospital, Aarhus, Denmark; ^3^Department of Clinical Medicine–Core Center for Molecular Morphology, Section for Stereology and Microscopy, Aarhus University, Aarhus, Denmark; ^4^Department of Neurology, Aarhus University Hospital, Aarhus, Denmark

## Abstract

**Background:**

Patients with Parkinson's disease (PD) often show peripheral autonomic dysfunction and depositions of pathological alpha-synuclein aggregates in the skin. However, functional consequences of this skin involvement have received little attention.

**Objective:**

To determine thermographic differences in the skin between healthy controls (HCs) and PD patients on hands, feet, and trunk and to correlate findings with symptoms and signs of dysautonomia. Between-group differences in autonomic parameters and questionnaires were explored.

**Methods:**

Twenty-one PD patients and 19 HCs were examined by thermographic infrared imaging of standardized anatomical locations on the trunk and upper and lower extremities at baseline and after exposure to cold stress test (CST). Thermal recovery rates (RRs) were determined on the basis of thermograms. Correlation analyses between alterations in skin temperature and autonomic dysfunction were performed.

**Results:**

The most significant RR difference between PD patients and HCs was seen on the fifth distal phalanx 10 minutes post-CST (mean RR ± SD: 51 ± 18% vs. 70 ± 23%, *p* = 0.003). No between-group differences were seen in baseline or post-CST values of the feet. No correlations were seen between thermal parameters and clinical and autonomic data. In the HC group, a positive, moderate correlation was seen between post-CST recovery values on the 3^rd^ and 5^th^ phalanx and body mass index (BMI) (*r* = 0.661, *p* = 0.002).

**Conclusions:**

The PD patients exhibited significant reduction in RR compared to HC and patients also displayed altered thermal responses in multiple anatomical locations. Thus, infrared thermography could become an important future tool in investigation of autonomic deficiency in PD.

## 1. Introduction

Parkinson's disease (PD) is a progressive neurodegenerative disease characterized by motor symptoms, including bradykinesia, tremor, and muscle rigidity [1]. The neuropathological hallmark of PD is intracellular protein inclusions of misfolded *α*-synuclein, termed Lewy bodies (LB) [2]. Lewy pathology is present in the central nervous system (CNS), but can also be found throughout the peripheral autonomic nervous system (PNS) [3] and has been detected in the enteric nervous system up to 20 years prior to diagnosis [4]. Symptoms of autonomic dysfunction including constipation, decreased saliva production, urinary dysfunction, orthostatic hypotension (OH), and sweating abnormalities are common in PD patients [5, 6].

Research in skin abnormalities in PD patients is gaining increasing interest, especially since Lewy pathology (aggregated alpha-synuclein) can be detected in autonomic nerve fibers in most patients using simple punch biopsies [7]. Also, a significant loss of autonomic nerve fibers, including fibers with vasomotor function, is seen in many patients [8], and it has been reported that PD patients have altered skin blood flow regulation resulting in cold limbs [9]. However, our understanding of autonomic skin dysfunction in PD is still very limited.

A recent study used infrared thermography and demonstrated that PD patients display abnormal thermal responses of the skin when exposed to cold stress test (CST), i.e., immersion of one hand in cold water and subsequent thermographic imaging of the recovery of skin temperature [10]. The authors reported reduced cooling and reduced thermal overshoot in the contralateral nonimmersed hand (NIH), as well as reduced recovery rates (RRs) in the immersed hand (IH) in the PD group compared to healthy controls (HCs). However, the authors only investigated thermographic data from the distal phalanx of the third finger [10], and the study has so far not been replicated.

The study aimed to determine thermographic differences in the skin post-CST between HC and PD patients on hands, feet, and trunk and to correlate findings with symptoms and signs of dysautonomia.

## 2. Materials and Methods

### 2.1. Ethics Statement

The study was approved by the Central Denmark Region Committee on Health Research Ethics (no. 1-10-72-277-16) and conducted in accordance with the Declaration of Helsinki. All participants provided written informed consent.

### 2.2. Subjects

In this cross-sectional study, 22 PD patients and 19 age- and sex-matched HCs were included between September and December 2017. PD patients fulfilled the diagnostic criteria of PD [11]. The PD patients were recruited from an earlier in-house project, while HCs were recruited via newspaper advertisement.

Inclusion criteria were as follows: age 50–90 years, ability to give informed consent, and fulfillment of diagnostic criteria (PD patients). Exclusion criteria were as follows: any neurological disease (except PD), significant medical disease of any kind (liver, kidney, heart, connective tissue, or endocrinological disease), known peripheral atherosclerotic disorder or intake of vasoregulatory substances (including catecholamines, phosphodiesterase inhibitors, calcium sensitizers, vasopressors, and digoxin), fever, infection in the past 14 days, and alcohol or drug abuse. Patients refrained from eating or drinking at least one hour prior to examinations. One PD patient was excluded after having eaten within 30 minutes before thermography. Two PD patients had restricted data due to technical issues, clinical findings (edema), and/or partial digit amputation. One PD patient was subsequently excluded, since he had eaten 30 min prior to thermography, leaving 21 PD patients in the study. The PD patients were studied on medication, and the L-dopa-equivalent daily dose (LEDD) was calculated.

### 2.3. Thermography

Infrared thermography was performed in all participants using a FLIR E60bx infrared camera (FLIR Systems, Inc. Wilsonville, Oregon, USA). A standardized examination setting was applied, which adhered closely to recommendations for infrared imaging in medicine [12, 13]. All subjects were studied in a 10 m^2^ darkened room between 9 am and 2 pm. All CSTs were performed with the patient sitting. Temperature and air humidity were monitored; room temperature was kept at 23 ± 1°C. The recorded air humidity was 55 ± 15%.

**Figure 1:**
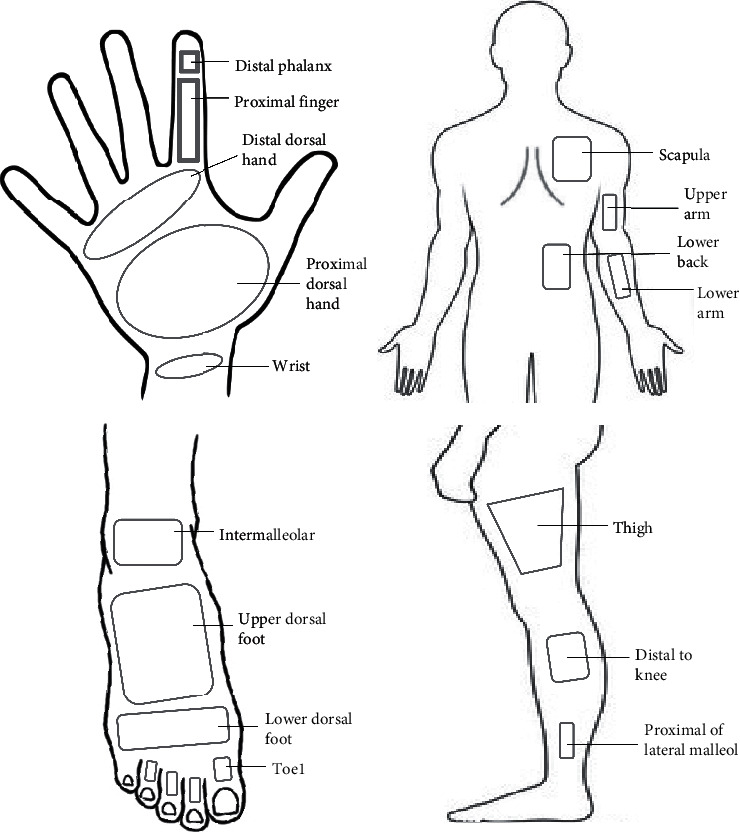
Schematic illustration of analyzed regions of interest (ROIs). Proportions are not accurate.

Three separate thermographic sessions were performed after acclimatization for at least 15 minutes and on the same day during a period of maximum 2 hours: (1) Regular anterior, posterior, and lateral (right and left) full-body images were acquired at baseline. (2) Baseline and post-CST images were acquired of the hands. CST was performed with the right hand immersed into cold water (3 ± 1°C) for two minutes. Water temperature was selected based on the only previous thermography study in patients with PD [10]. Water temperature was monitored using a digital thermometer prior to and during CST. Subsequently, images were obtained at 0, 2, 4, 6, 8, and 10 minutes postimmersion, comparable to previously published data [10]. (3) Baseline and post-CST images were acquired of the feet (procedure identical to CST of hands). Standardized regions of interest (ROIs) were used for analysis (Figure 1). During all three sessions, the camera was mounted on a tripod. Images were obtained with the patient placed at a standardized position in the room 300 cm (full body), 100 cm (hands), and 100 cm (feet) from the standardized position of the camera tripod. Standardized camera settings were applied: emissivity of 0.98 (human skin) [14], mean humidity of 55%, and mean reflected temperature of 20°C. There were no sources of airflow near or directly at the subject. Hands and feet were protected from direct contact with the cold water by a plastic glove/close-fitting plastic bag on the immersed hand/foot, respectively.

### 2.4. Clinical Assessment

Motor symptoms and disease stage of PD patients were scored using the MDS-UPDRS-III [15] and Hoehn & Yahr (H&Y) scales [16]. In all participants, blood pressure, distal blood pressure, and orthostatic hypotension test were performed. Distal blood pressures, measured by use of the strain gauge method on toes, served to exclude any subjects presenting abnormal values indicative of decreased peripheral blood pressure. Olfactory function was tested using the Sniffin' Sticks 16-item identification test [17]. Nonmotor symptoms were evaluated with the nonmotor symptoms questionnaire (NMSQuest) [18] and scales for outcome in Parkinson's disease—autonomic (SCOPA-AUT) [19]. Sleep behavior symptoms were scored with the REM sleep behavior disorder screening questionnaire (RBDSQ) [20], and cognition was evaluated with the Montreal Cognitive Assessment scale (MoCA) [21].

### 2.5. Data Analyses and Statistics

Thermographic images were analyzed using dedicated software, PMOD (PMOD Technologies LLC, Zürich, Switzerland). Analyses were performed blinded to clinical (PD/HC) category. For each hand (NIH and IH), 13 regions of interest (ROI) were defined (Figure 1). On the feet, 7 ROIs (immersed foot (IF) and nonimmersed foot (NIF)) were defined. On whole-body images at baseline, 3 ROIs on each leg/lateral side of the lower extremities and 4 ROIs on each upper extremity/back were defined.

To facilitate comparison to the study by Antonio-Rubio et al. [10], the skin of the dorsum of the third finger distal phalanx was included as a ROI. The main outcome was the calculated recovery rates (RRs) of the CSTs. These RRs were compared between groups and served as the main outcome when differentiating between groups. RRs were calculated as follows [10]:

(1) $$\begin{array}{c} \text{RR}=\frac{T_{x\text{post}}-T_{0\text{post}}}{T_{\text{baseline}}-T_{0\text{post}}}\cdot 100\%, \end{array}$$

in which *T* is the calibrated temperature (Celsius), and *x* indicates the time point of 2, 4, 6, 8, or 10 minutes postimmersion. Furthermore, thermal symmetry was calculated and equals the numeric value of the temperature difference between sides for each ROI.

Statistical analysis was carried out in GraphPad Prism (GraphPad Software, San Diego, CA, USA). For group comparisons, unpaired *t*-tests were applied for age, BMI, and baseline temperature data; Fisher's exact test was applied for sex (and other categorical variables), and nonparametric Mann–Whitney test was used for olfactory scores, MoCA, questionnaires, thermal symmetry, and RR. *Post hoc* statistical outlier analysis was applied to RR data. Post-CST group differences in RR over time were evaluated using two-way repeated-measures ANOVA. Group differences in RR were also interrogated using multivariate linear regression with correction for age, sex, and BMI. Correlations between thermal parameters and clinical parameters were interrogated using linear regression.

## 3. Results

**Figure 2:**
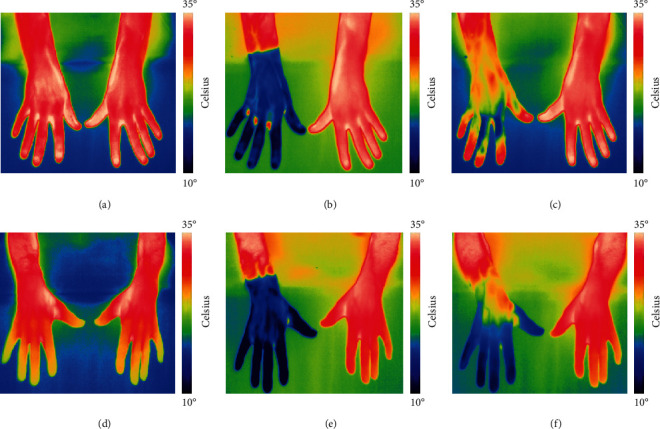
Thermographic images. A series of images from a healthy control ((a)–(c), RR = 75.0%) and a PD patient ((d)–(f), RR = 31.9%). Images at baseline (a, d), *T*_0_ postimmersion (b, e), and *T*_6_ postimmersion (c, f) are shown. All images are scaled 10–35°C. *Note.* The reduced recovery of temperature in the PD patient on the right immersed hand (IH).

Demographic data are listed in Table 1. The groups were well matched on sex and age, but the controls had slightly lower average BMI although not significant (*p* = 0.08). All participants were able to complete the CST. Representative thermal images of hands are shown in Figure 2. Clinical data and baseline thermography results are shown in Table 2.

At baseline, thermal asymmetry was seen only on the 1^st^ distal finger phalanx (more asymmetric in HC) and on the 2^nd^ toe and lower dorsal foot (more asymmetric in PD). In CST, after correcting for BMI, sex, and age and after excluding two significant outliers in the PD group (*p* = 0.0021; ROUT test, GraphPad Prism), significant between-group differences were seen on all five fingers at the *T*_6_, *T*_8_, and *T*_10_ time points, except for *T*_6_ on the 1^st^ and 3^rd^ fingers. The 5^th^ finger *T*_10_ RR was 27% lower in the PD group compared to the HC group (mean RR ± SD: 51 ± 18% vs. 70 ± 23%, *p* < 0.001).

**Figure 3:**
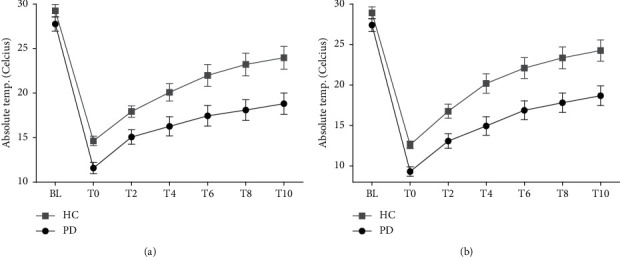
Cold stress test. Thermal recovery on the phalanx of the 3^rd^ finger (a) and the phalanx of the 5^th^ finger (b). Absolute temperature ± SEM.

Thermal recovery and differences between groups after hand CST are shown in Figure 3. Recovery is shown for the dorsal skin on the distal phalanx of the 3^rd^ finger for comparison with previously published results by Antonio-Rubio et al. [10], and on the 5^th^ distal phalanx dorsal skin, which showed the most significant between-group difference in our data.

No significant differences were seen in CST of the foot in any of the investigated ROIs (*p* > 0.05). The intermalleolar and upper foot ROIs were not analyzed, since the thermal images showed too large variance in water level and coverage of ROIs.

The RR showed positive correlation with BMI on the 3^rd^ and 5^th^ phalanx at all time points in the HC group (distal 5^th^ phalanx, 6 min, *p* = 0.002 *R*^2^ = 0.437), but not in the PD group. In the PD group, no significant correlations were seen between thermal baseline and CST parameters and age, UPDRS-III, H&Y, LEDD, SCOPA total score, RBDSQ score, or with diastolic or systolic blood pressure drops in the OH test (*p* > 0.05 in all tests).

## 4. Discussion

The study results show significantly altered thermal skin response in PD patients compared to healthy control subjects when exposed to CST, especially on the fingers. The decreased response showed increasing significance over time. At baseline, thermal asymmetry was seen only on the 1^st^ distal finger phalanx (more asymmetric in HC) and on the 2^nd^ toe and lower dorsal foot (more asymmetric in PD). No significant between-group differences were seen in baseline or post-CST values of the feet, and no correlations were seen between questionnaire and autonomic data and thermal responses in the PD group.

A previous study reported data from only the 3^rd^ finger distal phalanx, which showed decreased thermal recovery 6 min postimmersion in PD patients compared to HC (RR: 29 ± 17% vs. 55 ± 28%, *p* = 0.002) [10]. The recovery rates in our HC group were in general in agreement with this previous study (6 min post-CST, 3^rd^ finger 49%, 5^th^ finger 56%), whereas our PD cases seemed to show faster recovery (6 min post-CST, 3^rd^ finger 36%, 5^th^ finger 41%). Thus, our PD patients showed a more normal response compared to the previously studied group.

It is unclear what could have caused this difference, but our patients may have been at an earlier disease stage. They had shorter disease duration (4.8 y vs. 5.9 y), lower LEDD (315 vs. 619 mg), lower total SCOPA-AUT (13 vs. 18), and lower frequency of orthostatic hypotension (17% vs. 36%). Also, our HC group was carefully age-matched, whereas the HC group of the previous study was significantly younger (*p* = 0.04). In both studies, the thermography was carried out in the “on” state, so effects of dopaminergic replacement therapy probably did not affect the results. In summary, it is therefore possible that the previously reported thermal response to CST [10] applies to later stage PD patients, whereas the thermal response may be closer to normal at earlier stages of PD. Both the baseline and post-CST data showed considerable overlap between the PD and HC groups, and thermography therefore probably does not have diagnostic applicability—at least not with the currently implemented protocol. Of note, the recovery rate after CST varied slightly on different fingers. We do not have an explanation for this variation, and it could be a spurious finding.

The only published paper with PD patients only explored the hands after CST [10]. We wanted to exploratively study other regions. We had expected to see similar results on the CST of the feet, given that cold feet are a common complaint among PD patients and that the axons to the feet are longer than those innervating the hands. Also, previous studies have shown a drastic reduction of epidermal and piloerector nerve fibers to the distal lower extremities [22]. For these reasons, we wanted to obtain data from many different body regions to explore if other regions, including on the trunk and proximal extremities, were affected. Nevertheless, we did not see any difference in post-CST responses on the feet between PD and HC. We also did not see any thermal asymmetry on the proximal extremities and trunk ROIs, which is perhaps less surprising given that the density of epidermal, piloerector, and probably also arteriolar nerve fibers has found to be normal on the trunk of PD patients [22].

We found no correlation between baseline or post-CST thermal parameters and disease duration or motor symptom severity, LEDD, autonomic symptom burden (SCOPA-AUT), or the decline in blood pressure on tests of orthostatic hypotension. We only had three patients with OH, so it is possible that our patient sample was too healthy in this regard and that inclusion of a group of later stage cases with a higher prevalence of OH might have disclosed a significant association between thermal temperature responses and blood pressure regulation. Of note, limited correlation is also seen between MIBG heart scintigraphy values and OH parameters [6], suggesting that the cause of OH may be a complicated interplay between damage to the peripheral and central nervous systems.

This study has several limitations. The study sample size was only modest resulting in limited statistical power. However, the sample size is sufficient to conclude that thermography cannot reliably separate PD patients from controls at the individual level with the currently implemented protocol. It would have been preferable to have included a number of later stage PD patients with more progressed disease and involvement of the peripheral autonomic nervous system. The present study does not allow us to firmly conclude that thermal skin responses do not correlate with symptoms or other objective measures of autonomic dysfunction. It would also have been preferable to include skin biopsies with quantification of the amount of alpha-synuclein pathology as well as of the reduction in functional nerve fibers to arterioles. It is conceivable that a correlation may have been seen between skin pathology and denervation, assessed by histology, and the thermal skin responses. Finally, nutritional status can affect thermographic results of the skin. We did correct for BMI in our analyses, but more comprehensive indicators of nutritional status might be preferable.

## 5. Conclusion

We have shown that PD patients display an attenuated thermal recovery response to cold stress test of the hands but not of the feet, which agrees with one single previously published study. The thermal parameters did not show correlations with clinical or other autonomic parameters, which may have been caused in part by our inclusion of a fairly early stage population of PD patients. Thermography may have value as a research tool to study skin involvement in PD, and further studies should investigate the relationship between pathological thermal skin responses and autonomic denervation of the skin.

## Figures and Tables

**Table 1:** 

Demographics	PD patients (*n* = 21)	Healthy controls (*n* = 19)	*p* value
Age, years: mean ± SD (range)	66.7 ± 7.5 (52–84)	66.1 ± 5.4 (54–74)	0.8
Gender, M/F	13/8	12/7	>0.99
BMI, kg/m^2^: mean ± SD	26.6 ± 3.7	24.5 ± 3.5	0.080
UPDRS, median (range)	20 (8–33)	NA	NA
H&Y stage, I/II/III	7/12/2	NA	NA
Duration of disease, months: mean ± SD (range)	57.9 ± 34.8 (17–149)	NA	NA
Worse hemibody, right/left/symmetrical	13/8/0	NA	NA
LEDD, mg: median (range)	315 (15–1155)	NA	NA
SCOPA-AUT, mean ± SD	12.5 ± 6.8	3.6 ± 3.4	**<0.0001**
RBDSQ, mean ± SD	3.9 ± 3.1	0.7 ± 1.2	**0.0002**

NA: not available.

**Table 2:** 

Examinations, baseline	PD patients (*n* = 21)	Healthy controls (*n* = 19)	*p* value
Arterial hypertension, yes/no	11/10	9/10	>0.999
Orthostatic hypotension^1^, yes/no	3/18	5/14	0.442
Tympanic temperature, °C: mean ± SD	36.7 ± 0.3	36.6 ± 0.2	0.195
Subjective hyposmia, yes/no	19/2	2/17	**<0.0001**
Olfactory function, Sniffin' sticks; median (IQR)	7 (5.5–10)	12 (11–14)	**<0.0001**
Baseline temperature, 3^rd^ finger distal phalanx, °C: mean ± SD	27.8 ± 3.7	29.2 ± 3.1	0.1814
Baseline temperature, 5^th^ finger distal phalanx, °C: mean ± SD	27.4 ± 3.7	28.9 ± 3.2	0.1749
Thermal symmetry^2^, 1^st^ finger distal phalanx, °C: median (IQR)	0.22 (0.12–0.63)	0.52 (0.32–1.0)	**0.029**
Thermal symmetry^2^, lower dorsal foot, °C: median (IQR)	1.13 (0.49–1.98)	0.46 (0.33–0.69)	**0.002**
Thermal symmetry^2^, 2^nd^ toe, °C: median (IQR)	0.55 (0.32–1.89)	0.14 (0.03–0.63)	**0.012**

^1^Orthostatic hypotension: decline of ≥20/10 in mmHg in systolic/diastolic blood pressure. ^2^Thermal symmetry = temperature difference between contralateral ROIs, calculated from all ROIs on trunk/extremities. Only ROIs with significant baseline symmetry differences are listed.

## Data Availability

Data can be made available by contacting the corresponding author.

## Supplementary Materials

Supplementary Materials Supplementary Figure 1: recovery rate on hands in patients with Parkinson's disease (PD) and healthy control subjects (HC). Data are shown for each finger phalanx region at 4 (T4), 6 (T6), 8 (T8), and 10 (T10) minutes postimmersion. Only regions with significant between-group difference are shown.

## References

[1] Wolters E., Munter H., Steinbusch H., Wolters E., Baumann C. (2014). Parkinson’s disease. *Parkinson Disease and Other Movement Disorders*.

[2] Fearnley J. M., Lees A. J. (1991). Ageing and Parkinson’s disease: substantia nigra regional selectivity. *Brain*.

[3] Goedert M., Spillantini M. G., Del Tredici K., Braak H. (2013). 100 years of Lewy pathology. *Nature Reviews Neurology*.

[4] Stokholm M. G., Danielsen E. H., Hamilton-Dutoit S. J., Borghammer P. (2016). Pathological alpha-synuclein in gastrointestinal tissues from prodromal Parkinson’s disease patients. *Annals of Neurology*.

[5] Cersosimo M. G., Benarroch E. E. (2012). Autonomic involvement in Parkinson’s disease: pathology, pathophysiology, clinical features and possible peripheral biomarkers. *Journal of the Neurological Sciences*.

[6] Borghammer P., Knudsen K., Brooks D. J. (2016). Imaging systemic dysfunction in Parkinson’s disease. *Current Neurology and Neuroscience Reports*.

[7] Donadio V. (2018). Skin nerve alpha-synuclein deposits in Parkinson’s disease and other synucleinopathies: a review. *Clinical Autonomic Research*.

[8] Nolano M., Provitera V., Manganelli F. (2017). Loss of cutaneous large and small fibers in naive and l-dopa-treated PD patients. *Neurology*.

[9] Shindo K., Kobayashi F., Miwa M., Nagasaka T., Takiyama Y., Shiozawa Z. (2013). Temporal prolongation of decreased skin blood flow causes cold limbs in Parkinson’s disease. *Journal of Neural Transmission*.

[10] Antonio-Rubio I., Madrid-Navarro C. J., Salazar-López E. (2015). Abnormal thermography in Parkinson’s disease. *Parkinsonism & Related Disorders*.

[11] Postuma R. B., Berg D., Stern M. (2015). MDS clinical diagnostic criteria for Parkinson’s disease. *Movement Disorders*.

[12] Ring E. F. J., Ammer K. (2000). *The Technique of Infra Red Imaging in Medicine*.

[13] Moreira D. G., Costello J. T., Brito C. J. (2017). Thermographic imaging in sports and exercise medicine: a Delphi study and consensus statement on the measurement of human skin temperature. *Journal of Thermal Biology*.

[14] Ring E. F. J., Ammer K. (2012). Infrared thermal imaging in medicine. *Physiological Measurement*.

[15] Goetz C. G., Fahn S., Martinez-Martin P. (2007). Movement disorder society-sponsored revision of the unified Parkinson’s disease rating scale (MDS-UPDRS): process, format, and clinimetric testing plan. *Movement Disorders*.

[16] Hoehn M. M., Yahr M. D. (1967). Parkinsonism: onset, progression, and mortality. *Neurology*.

[17] Hummel T., Sekinger B., Wolf S. R., Pauli E., Kobal G. (1997). “Sniffin” Sticks’: olfactory performance assessed by the combined testing of odour identification, odor discrimination and olfactory threshold. *Chemical Senses*.

[18] Chaudhuri K. R., Martinez-Martin P., Schapira A. H. V. (2006). International multicenter pilot study of the first comprehensive self-completed nonmotor symptoms questionnaire for Parkinson’s disease: the NMSQuest study. *Movement Disorders*.

[19] Visser M., Marinus J., Stiggelbout A. M., Van Hilten J. J. (2004). Assessment of autonomic dysfunction in Parkinson’s disease: the SCOPA-AUT. *Movement Disorders*.

[20] Stiasny-Kolster K., Mayer G., Schäfer S., Möller J. C., Heinzel-Gutenbrunner M., Oertel W. H. (2007). The REM sleep behavior disorder screening questionnaire—a new diagnostic instrument. *Movement Disorders*.

[21] Nasreddine Z. S., Phillips N. A., BÃ©dirian V. r. (2005). The montreal cognitive assessment, MoCA: a brief screening tool for mild cognitive impairment. *Journal of the American Geriatrics Society*.

[22] Donadio V., Incensi A., Piccinini C. (2016). Skin nerve misfolded *α*-synuclein in pure autonomic failure and Parkinson disease. *Annals of Neurology*.

