# Supplementary material for: Skin Temperature in Parkinson's Disease Measured by Infrared Thermography
Source: Parkinsons Dis. 2020 Jul 25;2020:2349469. doi: 10.1155/2020/2349469 (PMC7397449; doi:10.1155/2020/2349469)
Supplement: Supplementary Materials — Supplementary Figure 1: recovery rate on hands in patients with Parkinson's disease (PD) and healthy control subjects (HC). Data are shown for each finger phalanx region at 4 (T4), 6 (T6), 8 (T8), and 10 (T10) minutes postimmersion. Only regions with significant between-group difference are shown. [file 2349469.f1.pdf]

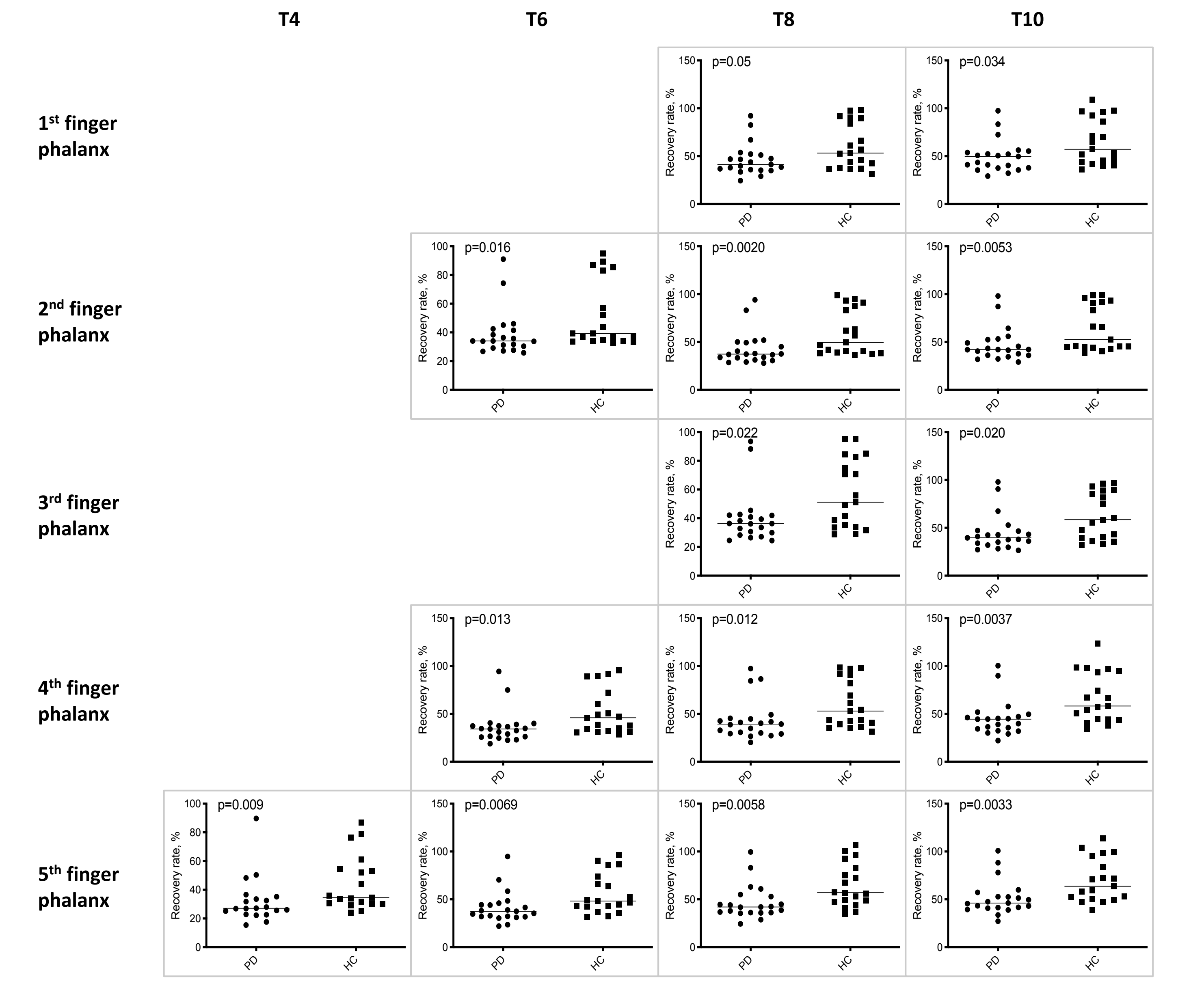

T4
T6
T8
T10
1st finger phalanx
2nd finger phalanx
3rd finger phalanx
4th finger phalanx
5th finger phalanx
p=0.05
p=0.034
p=0.016
p=0.0020
p=0.0053
p=0.022
p=0.020
p=0.013
p=0.012
p=0.0037
p=0.009
p=0.0069
p=0.0058
p=0.0033
Recovery rate, %
PD
HC
